# Fixed-Time Cooperative Formation Control of Heterogeneous Systems Under Multiple Constraints

**DOI:** 10.3390/e27050538

**Published:** 2025-05-17

**Authors:** Yandong Li, Wei Zhao, Ling Zhu, Zehua Zhang, Yuan Guo

**Affiliations:** 1 College of Computer and Control Engineering, Qiqihar University, Qiqihar 161000, China; liyandong@qqhru.edu.cn (Y.L.); woshiedward@163.com (Z.Z.); 2 Heilongjiang Key Laboratory of Big Data Network Security Detection and Analysis, Qiqihar University, Qiqihar 161000, China; 3 School of Mechanical and Electrical Engineering, Qiqihar University, Qiqihar 161000, China; 02652@qqhru.edu.cn; 4 School of Computer and Big Data, Heilongjiang University, Harbin 150080, China; guoyuan171@126.com

**Keywords:** UAV-UGV cooperative formation, fixed time, trajectory tracking, multiple constraints

## Abstract

This paper proposes a fixed-time formation-tracking control problem for a heterogeneous multi-agent system (MAS) consisting of six unmanned aerial vehicles (UAVs) and three unmanned ground vehicles (UGVs) under actuator attacks, external disturbances, and input saturation. First, a distributed sliding mode estimator and controller tailored for UAV-UGV heterogeneous systems are proposed based on sliding mode techniques. Second, by integrating repulsive potential functions with sliding manifolds, a distributed fixed-time adaptive sliding mode control protocol was designed. This protocol ensures collision avoidance while enabling the MASs to track desired trajectories and achieve a predefined formation configuration within a fixed time. The fixed-time stability of the closed-loop system was rigorously proven via Lyapunov theory.

## 1. Introduction

Heterogeneous MASs composed of unmanned ground vehicles (UGVs) and unmanned aerial vehicles (UAVs) integrate the advantages of UAVs for large-scale rapid reconnaissance and UGVs for precise ground target localization. Such systems have garnered increasing attention in both academic and practical domains [1,2,3]. However, ground–air cooperative formations face significant challenges. For example, practical systems inevitably suffer from the effects of input saturation, external disturbances, and actuator attacks. Moreover, as formation missions and heterogeneous systems grow in complexity, centralized control schemes become infeasible. Therefore, it is imperative to further explore effective distributed strategies.

The coordinated control field has seen substantial research focus on MASs’ formation tracking challenges, a critical subset of multi-agent coordination. Formation tracking control requires followers to maintain predefined formation configurations while tracking the leader’s states. In the domain of air–ground cooperation, several classical formation tracking approaches have been proposed, including leader–follower distributed control [4,5], fault-tolerant control [6], observer design [7,8,9], and integrated adaptive control with sliding mode control [10,11]. These methodologies enable followers to achieve trajectory tracking through specifically designed controllers. For instance, reference [7] investigated distributed fixed-time formation-tracking control for nonholonomic wheeled mobile robot systems under directed fixed/switching topologies. It proposed a control protocol based on distributed observers, which transforms the formation problem into a consensus control problem, ensuring followers track the leader and establish desired formations within a fixed time. Reference [11] addressed second-order MASs by integrating repulsive potential functions with sliding manifolds, developing a distributed fixed-time sliding mode adaptive formation control strategy. This approach achieves fixed-time formation tracking while realizing collision avoidance between agents under undirected connected topologies.

The convergence rate serves as a critical performance metric in formation control studies, particularly for achieving predefined multi-agent configurations within finite-time horizons. For instance, heterogeneous multi-agent systems comprising UGVs and UAVs must accomplish formation missions under strict temporal constraints [12,13,14]. Nevertheless, the settling time of finite-time coordination protocols is generally influenced by the agents’ initial states. In scenarios involving excessively large or unavailable initial conditions, convergence within prescribed deadlines may prove unattainable. Moreover, even with accessible initialization parameters, specific values could induce prolonged settling durations, thereby constraining the applicability of finite-time consensus frameworks. To circumvent this limitation, the notion of fixed-time stability has been incorporated [15], offering an explicit upper-bound convergence time independent of the initial state information. Consequently, fixed-time cooperative control strategies for networked multi-agent systems have attracted substantial research interest in recent years [16,17,18].

In practical formation-tracking control tasks for MASs, critical real-world challenges, such as input saturation, external disturbances, and actuator attacks, must be rigorously addressed to ensure robustness and closed-loop stability in dynamic environments. For instance, Vazquez Trejo, J. A. et al. [19] proposed a fault-tolerant consensus control method for continuous-time multi-agent systems subject to additive and multiplicative actuator faults by utilizing distributed LPV observers and virtual actuators to redistribute control inputs. Wang Lei Cheng et al. [20] proposed a neural network-based adaptive nonsingular fast terminal sliding mode formation control method for heterogeneous UGV-UAV systems subject to actuator faults, model uncertainties, and external disturbances, achieving finite-time convergence of heterogeneous formation errors. Addressing similar issues, Wang Lei Cheng et al. [21] developed a fixed-time formation-tracking control framework leveraging fixed-time distributed observers, disturbance observers, and backstepping techniques to ensure system performance. Hao Xiong et al. [22] designed a distributed formation-tracking strategy for heterogeneous MASs under external disturbances and model uncertainties, integrating adaptive fractional-order sliding mode control with feedback multilayer fuzzy neural networks. Their approach incorporated an event-triggered mechanism to reduce the communication frequency and validated the robustness and stability of the system under uncertainties. Although studies [20,21,22] addressed practical constraints in heterogeneous multi-agent systems, they neglected the collision avoidance problem between agents.

Adderson et al. [23] proposed a time-varying formation control method for heterogeneous MASs based on an improved artificial potential field (APF) algorithm. By integrating agent motion state parameters to optimize obstacle-avoidance paths and employing terminal sliding mode controllers, they achieved simulation and experimental validations of hybrid formations involving quadrotors and mobile robots. Under the constraints of collision avoidance, actuator attacks, and Markov switching topologies, Xisheng Zhan et al. [24] studied the fixed-time formation control problem involving multiple groups with time-varying configurations in second-order nonlinear multi-agent systems. They developed a distributed fixed-time sliding mode manifold control strategy using RBF neural networks (to approximate unknown nonlinear terms), enabling collision-avoidance formations under switching topologies. While references [23,24] adopted generic nonlinear models that rely solely on the relative position or distance control; such frameworks struggle to eliminate rotational degrees of freedom in formations, potentially leading to non-rigid morphologies. In contrast, practical systems (e.g., UAVs, intelligent vehicles) are often constrained by kinematic models with limitations on steering angles and heading angles. This work utilized a nonholonomic constrained model to achieve directional coordination by adjusting the heading angles during obstacle avoidance or trajectory tracking, thereby avoiding control saturation or oscillations caused by steering constraints.

Building upon existing research in fixed-time formation tracking, collision avoidance, and multi-constraint handling, this study further investigated the fixed-time coordinated formation control problem for heterogeneous systems under multiple constraints. The main contributions are summarized as follows:

1. Multi-constraint integration: This work addresses practical challenges in formation control, including input saturation, external disturbances, and actuator attacks, by introducing a fixed-time control framework independent of the initial system states. This ensures the fixed-time convergence of formation tracking errors under these constraints.
2. For the collision avoidance problem in multi-unmanned vehicle formation processes, a control strategy was designed by integrating sliding mode controllers with the artificial potential field method, which achieved collision avoidance between multiple agents while ensuring robustness and real-time performance in dynamic obstacle avoidance.
3. Distributed fixed-time estimation and tracking: To achieve the fixed-time stable tracking of UAVs toward a virtual leader UGV, for each follower, a distributed fixed-time sliding mode estimator was developed. This estimator reconstructs the virtual leader’s state information within a fixed time. Subsequently, a formation controller is designed based on trajectory tracking errors, enabling UAVs to rapidly form a reconnaissance formation in a fixed time.

The remainder of this paper is organized as follows: Section 2 introduces preliminary knowledge and presents relevant lemmas. Section 3 establishes the dynamic models for both UAVs and UGVs. In Section 4, the design of a formation controller is described for each follower UAV based on its estimation of the virtual leader’s state, while an adaptive fixed-time controller is developed for each follower UGV to address multi-vehicle formation collision avoidance under external disturbances, actuator attacks, and input saturation, with stability proofs provided for both controllers. Section 5 presents the results of simulation experiments conducted to verify the effectiveness of the control protocol and the feasibility of the control strategy. Finally, Section 6 summarizes the core content of this paper.

## 2. Graph Theory and Related Lemmas

### 2.1. Graph Theory

For a heterogeneous formation system, the communication topology between followers is described by an undirected graph G={W,E,A}, where $W=\left\{\begin{array}{c} w_{1},w_{2},\ldots ,w_{n} \end{array}\right\}$ denotes the node set, E∈W×W represents the edge set, and $A=\left[\begin{array}{c} a_{ij} \end{array}\right]\in R^{n\times n}$ is the adjacency matrix of G. An edge $(w_{i},w_{j})\in W$ signifies that agents $w_{j}$ and $w_{i}$ can exchange information bidirectionally. The degree matrix of graph G is denoted as $D=diag\left\{\begin{array}{c} d_{1},d_{2},\ldots ,d_{n} \end{array}\right\}$, where $d_{i}=\sum_{j=1}^{n}a_{ij},i=1,2,\ldots n$. The Laplacian matrix $L=\left[l_{ij}\right]\in R^{n\times n}$ is defined as L=D−A. The leader adjacency matrix can be expressed as a diagonal matrix $B=diag\left\{\begin{array}{c} b_{1},b_{2},\ldots ,b_{n} \end{array}\right\}\in R^{n\times n}$, where $b_{i}>0$ indicates that follower i can access the leader’s information; otherwise, $b_{i}=0$.

### 2.2. Related Lemmas for Multi-Agent Systems

**Lemma 1**  ([15,25])**.** *Consider the system*

(1) $$\dot{x}=f\left(x\right),f\left(0\right)=0,x\in R^{n}$$

There exists a continuous function V(x) fulfilling the conditions enumerated below:

1. V(x) is positive definite;
2. There are scalars a>0, b>0, and c∈(1,2) such that

(2) $$\dot{V}\left(x\right)+aV^{c}\left(x\right)+bV^{2-c}\left(x\right)\leq 0$$

Therefore, the origin serves as a fixed-time stable equilibrium point for system (1). Moreover, the convergence time is given by Equation (3):

(3) $$T\leq \frac{\pi }{2\sqrt{ab(c-1)}}$$

**Lemma 2**  ([26])**.** *For a connected undirected network topology, if at least one follower agent can access the leader’s state information, the matrix H (defined as H = L + B) is symmetric and positive definite.*

**Lemma 3**  ([15,25])**.** *Provided that a Lyapunov function V(x) is constructed for the multi-agent system ensuring*

(4) $$\dot{V}\left(x\right)\leq -\alpha V^{p}\left(x\right)-\beta V^{q}\left(x\right)+\eta$$

where η>0, it satisfies the following conditions:

(5) $$x\in \left\{V\left(x\right)\leq \min\left\{{\left(\frac{\eta }{(1-\varpi )\alpha }\right)}^{\frac{1}{p}},{\left(\frac{\eta }{(1-\varpi )\beta }\right)}^{\frac{1}{q}}\right\}\right\}$$

where 0<ϖ<1, and the fixed-time stability of system (1) is ensured, where the convergence time satisfies T:

(6) $$T\leq T_{\max}:=\frac{1}{\alpha \varpi \left(p-1\right)}+\frac{1}{\beta \varpi \left(1-q\right)}$$

**Lemma 4**  ([27])**.** *For any $x_{1},x_{2},\ldots ,x_{n}\geq 0$, the following inequality holds:*

(7) $$\left\{\begin{array}{cc} {\left(\sum_{i=1}^{n}x_{i}\right)}^{p}\leq \sum_{i=1}^{n}x_{i}^{p}, & \mathrm{if}\;0<p\leq 1,\\ n^{1-p}{\left(\sum_{i=1}^{n}x_{i}\right)}^{p}\leq \sum_{i=1}^{n}x_{i}^{p}, & \mathrm{if}\;p>1. \end{array}\right.$$

## 3. System Modeling

### 3.1. Kinematic Model of UAV

**Figure 1:**
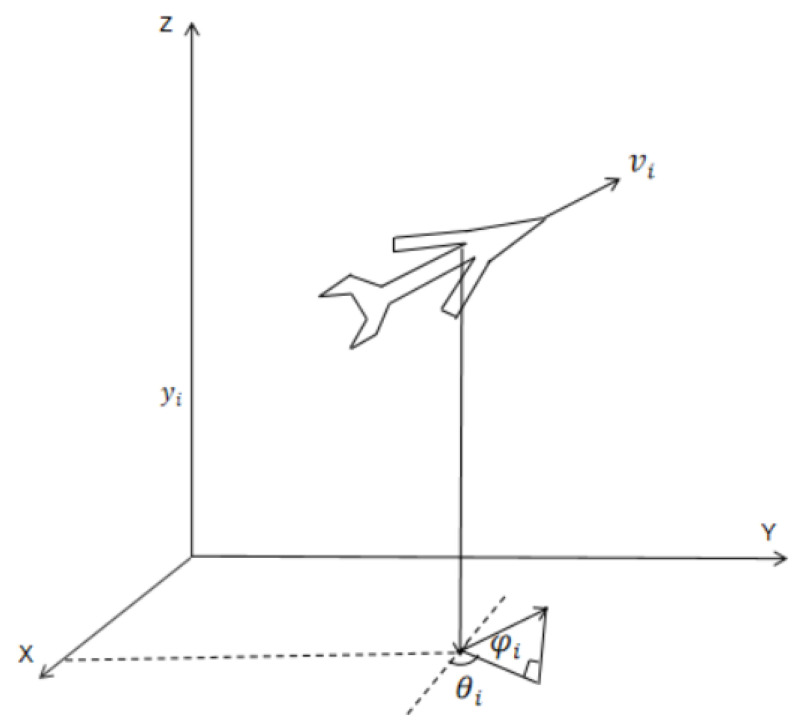
UAV system model.

Figure 1 presents the model of the UAV. The UAV’s kinematic model considers only the yaw angle $\theta_{i}$ and pitch angle $\varphi_{i}$ in its formulation.

The control problem of the UAV studied in this paper primarily focuses on the position system, and its kinematic equations can be formulated as [28]

(8) $$\begin{array}{cc} {\dot{x}}_{i} & =\nu_{i}\cos\left(\theta_{i}\right)\cos\left(\phi_{i}\right)\\ {\dot{y}}_{i} & =\nu_{i}\sin\left(\theta_{i}\right)\cos\left(\phi_{i}\right)\\ {\dot{z}}_{i} & =\nu_{i}\sin\left(\phi_{i}\right) \end{array}$$

where $x_{i}$, $y_{i}$, and $z_{i}$ represent the UAV’s position information; $v_{i}$ denotes the UAV’s ground speed; $\theta_{i}$ is the pitch angle for the UAV agent; and $\varphi_{i}$ is the yaw angle for the UAV agent.

### 3.2. Dynamic Model of UGV

Given that the UGV system accounts for input saturation, actuator attacks, and external disturbances, the variable ${\ddot{x}}_{gi}$ can be designed as follows:

(9) $${\ddot{x}}_{gi}\left(t\right)=sat\left(u_{gi}\left(t\right)\right)+q_{i}\left(t\right)\phi \left(x_{gi,1},x_{gi,2},t\right)+∋\left(X_{gi},t\right)$$

Let

(10) $$\left\{\begin{array}{c} x_{gi,1}=x_{gi}\\ x_{gi,2}={\dot{x}}_{gi} \end{array}\right.$$

From Equations (9) and (10), it follows that

(11) $$\left\{\begin{array}{c} \begin{array}{cc} {\dot{x}}_{gi,1} & =x_{gi,2}\\ {\dot{x}}_{gi,2} & =\mathrm{sat}\left(u_{gi}\left(t\right)\right)+q_{i}\left(t\right)\phi \left(x_{gi,1},x_{gi,2},t\right)+∋\left(X_{gi},t\right) \end{array} \end{array}\right.$$

where $x_{gi,1}\left(t\right)$ and $x_{gi,2}\left(t\right)$ denote the position and velocity, $u_{gi}\left(t\right)$ represents the control input, $sat(u_{gi}\left(t\right))$ denotes the input saturation function, $∋(X_{gi},t)$ represents the disturbance acting, and $\phi (x_{gi,1},x_{gi,2},t)$ is a continuously differentiable unknown function. When subjected to actuator attacks, $q_{i}\left(t\right)=1$; otherwise, $q_{i}\left(t\right)=0$.

**Remark 1.**  *Since UAVs operate in three-dimensional space while UGVs move within a two-dimensional plane, only horizontal position information needs to be exchanged between the UAVs and the leading UGV during the formation configuration.*

**Remark 2.**  *The formation control objective for the UAV-UGV is to ensure that UAVs track the trajectory of the leading UGV, while the UAVs and UGVs need to achieve predefined formation geometries within a fixed time.*

## 4. Controller Design and Stability Analysis

For heterogeneous UAV-UGV multi-agent systems subject to external disturbances, actuator attacks, and input saturation, this work proposes a distributed fixed-time sliding mode estimator and controller that incorporates collision avoidance between agents. The designed framework ensures that each follower achieves trajectory-tracking control of the leader and completes the formation within a fixed time, with all closed-loop signals converging to a small neighborhood of zero within a bounded time interval.

**Assumption 1.**  *The unknown disturbances $∋(X_{gi},t)$ and the leader’s control input are bounded such that $\left|∋(X_{gi},t)\right|\leq \sigma$ and $\|u_{0(t)}\|\leq u_{b}$, where σ>0 and $u_{b}>0$.*

**Assumption 2**  ([11])**.** *The undirected graph G is connected, and one or more followers can access the leader’s state.*

**Assumption 3.**  *The desired position trajectory $X_{0}$, ${\dot{X}}_{0}$, and ${\ddot{X}}_{0}$ of the leader is bounded.*

### 4.1. Fixed-Time Formation Control for UAVs

The purpose of formation control is to enable the UAVs system to achieve desired collective motion in a predefined formation pattern. The control objectives of heterogeneous UAV systems can be formulated as

(12) $$\left\{\begin{array}{cc} \lim_{t\to T_{ix}}\left(x_{i}\left(t\right)-x_{0}\left(t\right)\right) & =\sigma_{ix},\\ \lim_{t\to T_{iy}}\left(y_{i}\left(t\right)-y_{0}\left(t\right)\right) & =\sigma_{iy},\\ \lim_{t\to T_{i\theta }}\left(\theta_{i}\left(t\right)-\theta_{0}\left(t\right)\right) & =\sigma_{i\theta }. \end{array}\right.$$

where $\sigma_{i}=\left(\sigma_{ix},\sigma_{iy},\sigma_{i\theta }\right)$ represents the desired attitude of the UAV relative to the leader.

#### 4.1.1. Distributed Fixed-Time Sliding Mode Estimator

The state of the leader is represented as $\xi_{0}={\left[x_{0},y_{0},\theta_{0},\nu_{0},w_{0}\right]}^{T}$. The estimated state for the follower relative to the leader is formulated as ${\hat{\xi }}_{i}={\left[{\hat{x}}_{i},{\hat{y}}_{i},{\hat{\theta }}_{i},{\hat{v}}_{i},{\hat{w}}_{i}\right]}^{T}$. We propose a distributed fixed-time sliding mode estimator to estimate the state information of $\xi_{0}$, where for $b_{i}>0$, followers have direct access to the leader’s information without requiring an estimator.

The attitude tracking error can be formulated as

(13) $$\left\{\begin{array}{cc} e_{ix} & =\sum_{j=1}^{N}a_{ij}\left({\hat{x}}_{i}-{\hat{x}}_{j}\right)+b_{i}\left({\hat{x}}_{j}-x_{0}\right),\\ e_{iy} & =\sum_{j=1}^{N}a_{ij}\left({\hat{y}}_{i}-{\hat{y}}_{j}\right)+b_{i}\left({\hat{y}}_{j}-y_{0}\right),\\ e_{i\theta } & =\sum_{j=1}^{N}a_{ij}\left({\hat{\theta }}_{i}-{\hat{\theta }}_{j}\right)+b_{i}\left({\hat{\theta }}_{j}-\theta_{0}\right). \end{array}\right.$$

The estimation system can be designed based on the attitude tracking error as

(14) $${\dot{\hat{x}}}_{i}=\frac{d_{i}{\dot{\hat{x}}}_{j}+b_{i}{\dot{x}}_{0}-C_{x}sign\left(e_{ix}\right){\left(e_{ix}\right)}^{1-\frac{1}{m}}-D_{x}sig{\left(e_{ix}\right)}^{1+\frac{1}{m}}}{d_{i}+b_{i}}$$

(15) $${\dot{\hat{y}}}_{i}=\frac{d_{i}{\dot{\hat{y}}}_{j}+b_{i}{\dot{y}}_{0}-C_{y}sign\left(e_{iy}\right){\left(e_{iy}\right)}^{1-\frac{1}{m}}-D_{y}sig{\left(e_{iy}\right)}^{1+\frac{1}{m}}}{d_{i}+b_{i}}$$

(16) $${\dot{\hat{\theta }}}_{i}=\frac{d_{i}{\dot{\hat{\theta }}}_{j}+b_{i}{\dot{\theta }}_{0}-C_{\theta }sign\left(e_{i\theta }\right){\left(e_{i\theta }\right)}^{1-\frac{1}{m}}-D_{\theta }sig{\left(e_{i\theta }\right)}^{1+\frac{1}{m}}}{d_{i}+b_{i}}$$

where $C_{x}>0,D_{x}>0$, m∈(0,1], ${\mathrm{sig}}^{\alpha }\left(x\right)={\left|x\right|}^{\alpha }\mathrm{sign}\left(x\right)$, and sign(.) is a symbolic function.

**Theorem 1.**  *For system (8) under the distributed fixed-time sliding mode estimation laws (14)–(16), if $\lim_{t\to T_{\mathrm{ix}}}e_{ix}=0$ is implemented, then the follower UAVs achieve fixed-time tracking of the virtual leader such that ${\hat{x}}_{i}=x_{0}.$*

**Proof of Theorem 1.**  Combining Equations (13) and (14), we conclude that

(17) $${\dot{e}}_{ix}=-C_{x}sign\left(e_{ix}\right){\left(e_{ix}\right)}^{1-\frac{1}{m}}-D_{x}sig{\left(e_{ix}\right)}^{1+\frac{1}{m}}$$

The Lyapunov function is introduced as

(18) $$V_{1}=\frac{1}{2}e_{ix}^{2}$$

Taking the derivative of $V_{1}$ yields

(19) $$\begin{array}{cc} \dot{V_{1}} & =-C_{x}|e_{ix}{|}^{2-\frac{1}{m}}-D_{x}|e_{ix}{|}^{2+\frac{1}{m}}\leq 0\\ & \Rightarrow {\dot{V}}_{ix}+C_{x}2^{1-\frac{1}{2m}}V_{ix}^{1-\frac{1}{2m}}+D_{x}2^{1+\frac{1}{2m}}V_{ix}^{1+\frac{1}{2m}}\leq 0 \end{array}$$

The system satisfies the fixed-time convergence condition under multi-agent coordination. Then, according to Lemma 1, the settling time can be derived as $T_{ix}$:

(20) $$T_{ix}\leq \frac{\pi }{2\sqrt{2^{2}C_{X}D_{x}\left(1+\frac{1}{2m}-1\right)}}=\frac{\pi }{2\sqrt{\frac{2}{m}C_{X}D_{x}}}$$

Therefore, it can be concluded that $e_{ix}$ asymptotically converges to a small neighborhood around zero within a fixed and tunable time horizon:

(21) $${\left[\begin{array}{c} e_{1x}\ldots e_{nx} \end{array}\right]}^{T}=\left(L+B\right){\left[\begin{array}{c} {\hat{x}}_{i}-x_{0}....{\hat{x}}_{n}-x_{0} \end{array}\right]}^{T}$$

According to Assumption (2), since the matrix H=L+B>0, it follows that H is positive definite. From Equation (21), it follows that ${\hat{x}}_{i}=x_{0}$, which implies that the system achieves fixed-time stability. □

Similarly, for estimators (15) and (16), it holds that ${\hat{y}}_{i}=y_{0}$ and ${\hat{\theta }}_{i}\;=\;\theta_{0}$ within a fixed time. The convergence times $T_{\mathrm{iy}}$ and $T_{i\theta }$ can be derived:

(22) $$T_{iy}\leq \frac{\pi }{2\sqrt{\frac{2}{m}C_{y}D_{y}}}$$

(23) $$T_{i\theta }\leq \frac{\pi }{2\sqrt{\frac{2}{m}C_{\theta }D_{\theta }}}$$

As demonstrated above, all attitude errors $e_{ix}$, $e_{iy}$, and $e_{i\theta }$ converge to the equilibrium point within a fixed and adjustable time $T_{s}<\max\left\{\begin{array}{c} T_{ix},T_{iy},T_{i\theta } \end{array}\right\}$.

Based on the aforementioned estimation system, the estimator can be constructed as

(24) $${\hat{w}}_{i}={\dot{\hat{\theta }}}_{i}$$

(25) $${\hat{\nu }}_{i}=\mathrm{sgn}\left({\dot{\hat{x}}}_{i}\cos{\hat{\theta }}_{i}\right){\left({\dot{\hat{x}}}_{i}^{2}+{\dot{\hat{y}}}_{i}^{2}\right)}^{\frac{1}{2}}$$

where *w* is the yaw angular velocity.

#### 4.1.2. Controller Design

The tracking error of the follower UAV(s) can be formulated as

(26) $$z_{ix}=x_{i}\left(t\right)-{\hat{x}}_{i}\left(t\right)-\sigma_{ix}$$

(27) $$z_{iy}=y_{i}\left(t\right)-{\hat{y}}_{i}\left(t\right)-\sigma_{iy}$$

(28) $$z_{i\theta }=\theta_{i}\left(t\right)-{\hat{\theta }}_{i}\left(t\right)-\sigma_{i\theta }$$

where $z_{i}={\left[z_{ix},z_{iy,}z_{i\theta }\right]}^{T}$. The global coordinate errors can be transformed into local coordinate errors via the coordinate transformation matrix:

(29) $$z_{i1}=\left[\begin{array}{c} x_{ie}\\ y_{ie}\\ \theta_{ie} \end{array}\right]=\left[\begin{array}{ccc} \cos\theta_{i} & \sin\theta_{i} & 0\\ -\sin\theta_{i} & \cos\theta_{i} & 0\\ 0 & 0 & 1 \end{array}\right]\left[\begin{array}{c} z_{ix}\\ z_{iy}\\ z_{i\theta } \end{array}\right]$$

By taking the derivative of Equation (29), the following can be obtained:

(30) $${\dot{x}}_{ie}=w_{i}y_{ie}+\nu_{i}-{\hat{\nu }}_{i}\cos\theta_{ie}$$

(31) $${\dot{y}}_{ie}=-w_{i}x_{ie}+{\hat{\nu }}_{i}\sin\theta_{ie}$$

(32) $${\dot{\theta }}_{ie}=w_{i}-{\hat{w}}_{i}$$

Based on the aforementioned error dynamics of the UAVs, we designed the formation control laws for the follower UAVs as

(33) $$\nu_{i}={\hat{\nu }}_{i}\cos\theta_{ie}-\mu_{i1}\tanh x_{ie}$$

(34) $$w_{i}={\hat{w}}_{i}-\mu_{i2}\frac{y_{ie}\sin\theta_{ie}}{\left(1+x_{ie}^{2}+y_{ie}^{2}\right)\theta_{ie}}{\hat{\nu }}_{i}-\mu_{i3}sig^{\mu_{i4}}\theta_{ie}$$

where $\mu_{\mathrm{ij}}>0,j=1,2,3,4$.

### 4.2. Fixed-Time Formation Control of UGVs

The objective of multi-unmanned vehicle formation control is to establish a robust fixed-time algorithm that considers collision avoidance between the agents under the presence of external disturbances, actuator attacks, and input saturation constraints. This algorithm ensures that the multi-unmanned vehicles achieve the desired formation configuration, where $x_{gi}\to x_{gi}^{d}$ and $x_{gi}-x_{gj}\to \Delta_{ij}$ with bounded tracking errors, while avoiding collisions during formation tracking, where $\|x_{gi}-x_{gj}\|>R_{in}$.

#### 4.2.1. Fixed-Time Sliding Manifold Design

For the collision avoidance problem of agents in a UGV system, two circular regions are introduced: the collision avoidance region $\Omega_{ci}=\left\{\begin{array}{c} x_{gj}\left\|x_{gi}-x_{gj}\right\|\leq R_{in} \end{array}\right\}$ and the detection region $\Omega_{di}=\left\{x_{gj}\left\|x_{gi}-x_{gj}\right\|\leq R_{out}\right\}$, where $R_{in}$ and $R_{out}$ denote the radii, where $0<R_{in}<R_{out}$. The repulsive potential function (RPF) can then be designed as follows:

(35) $$\begin{array}{c} J_{gij}\left(\right\|x_{gi}-x_{gj}\left\|\right)=\left\{\begin{array}{cc} \rho_{ij}\frac{(R_{\mathrm{out}}^{2}-\|x_{gi}-x_{gj}{{\|}^{2})}^{2}}{\left(\right\|x_{gi}-x_{gj}{{\|}^{2}-R_{m}^{2})}^{2}},\; & \mathrm{if}\;R_{\mathrm{in}}\leq \left\|x_{gi}-x_{gj}\right\|\leq R_{\mathrm{out}},\\ 0,\; & \mathrm{if}\;\|x_{gi}-x_{gj}\|\;\geq \;R_{\mathrm{out}}. \end{array}\right. \end{array}$$

where $\rho_{ij}$ is a positive constant. The following conclusion can be drawn from Equation (35): if the distance between the j-th follower UGV and the i-th follower UGV exceeds radius $R_{out}$, the potential function satisfies $J_{gij}$ equal to zero; if the distance equals $R_{in}$, the potential function $J_{gij}$ asymptotically approaches infinity.

The formation geometry of the UGVs is determined by the formation shape parameter $\Delta_{ij}\in R$, where $\Delta_{ij}$ denotes the desired positional deviation between the *i*-th UGV and the *j*-th UGV in the multi-agent system:

(36) $$\Delta_{ij}=\Delta_{i}-\Delta_{j}$$

where $\Delta_{i}\in R^{m}$ denotes the desired positional deviation of the i-th follower UGV relative to the leader in the multi-agent system. Therefore, $x_{gi}^{d}=x_{0}+\Delta_{i}\in R^{m}$ denotes the follower’s expected position, and $\nu_{gi}^{d}=\nu_{0}\in R^{m}$ denotes the follower’s expected velocity, where $x_{0}\left(t\right)$ is the leader’s position and $v_{0}\left(t\right)$ is the leader’s velocity of the formation center.

**Assumption 4.**  *Collision avoidance between agents is inherently guaranteed upon convergence to the desired formation geometry, where $∥\Delta_{ij}∥>R_{in}$.*

The trajectory tracking error of the UGV can be formulated as

(37) $$\left\{\begin{array}{c} e_{gi,1}=\sum_{j=1}^{n}a_{ij}\left(x_{gi}-x_{gj}-\Delta_{ij}\right)+b_{i}\left(x_{gi}-x_{gi}^{d}\right)\\ e_{gi,2}=\sum_{j=1}^{n}a_{ij}\left(x_{gi,2}-x_{gj,2}-\Delta_{ij}\right)+b_{i}\left(x_{gi,2}-\nu_{0}\right) \end{array}\right.$$

where $e_{gi,1}={\left[e_{xgi,1},e_{ygi,1}\right]}^{T}$ and $e_{gi,2}={\left[e_{xgi,2},e_{ygi,2}\right]}^{T}$. $X_{0}$ and ${\dot{X}}_{0}$ are the expected position and velocity of the UGV, respectively; $X_{gi,1}$ and $X_{gi,2}$ are the position and velocity of the UGV, respectively.

The following fixed-time sliding manifold is developed:

(38) $$s_{gi}=K_{1}\;\mathrm{Tanh}\left(e_{gi,2}\right)+g_{i}{\mathrm{sig}}^{\mu_{1}}\left(e_{gi,1}\right)+g_{2}h\left(e_{gi,1}\right)$$

where $g_{i}=g_{0}+\sum_{j\neq 1,j\neq i}^{n}J_{ij}\left(\right\|x_{i}-x_{j}\left\|\right),K_{1}>0,g_{0}>0,g_{2}>0,\mu_{1}=\frac{m_{1}}{n_{1}}$, and $m_{1}$ and $n_{1}$ are two odd integers with $m_{1}>n_{1}>0.$

(39) $$\mathrm{Tanh}\left(e_{gi,2}\right)={\left[\mathrm{Tanh}\left(e_{xgi,2}\right),\mathrm{Tanh}\left(e_{ygi,2}\right)\right]}^{T}$$

(40) $$h\left(e_{gi,1}\right)={\left[h\left(e_{xgi,1}\right),h\left(e_{ygi,1}\right)\right]}^{T}$$

(41) $$h\left(e_{g_{i,1}}\right)=\left\{\begin{array}{cc} {\left|e_{g_{i,1}}\right|}^{\mu_{2}}\mathrm{sign}\left(e_{g_{i,1}}\right), & \mathrm{if}\;|e_{g_{i,1}}|>\delta \\ \left|ae_{g_{i,1}}+b\right|{\left|e_{g_{i,1}}\right|}^{\nu_{0}}\mathrm{sign}\left(e_{g_{i,1}}\right), & \mathrm{if}\;|e_{g_{i,1}}|\leq \delta \end{array}\right.$$

where $\mu_{2}=\frac{m_{2}}{n_{2}},\;m_{2}$ and $n_{2}$ are two odd integers with $m_{2}>n_{2}>0,\;1<r_{0}<2,$ $a=\frac{r_{0}-\mu_{2}}{r_{0}-1}\delta^{\mu_{2}-1},\;b=\frac{1-\mu_{2}}{1-r_{0}}\delta^{\mu_{2}-r_{0}}$, and δ is a small positive constant.

#### 4.2.2. Distributed Sliding Mode Controller Design

By taking the derivative of $e_{gi,2}$, we can obtain

(42) $${\dot{e}}_{gi,2}=\sum_{j=1}^{n}a_{ij}\left({\dot{x}}_{gi,2}-{\dot{x}}_{gj,2}\right)+b_{i}\left({\dot{x}}_{gi,2}-{\dot{\nu }}_{0}\right)$$

Substituting Equation (11) into Equation (42) yields

(43) $$\begin{array}{cc} {\dot{e}}_{gi,2} & =\sum_{j=1}^{n}a_{ij}\left(\mathrm{sat}\left(u_{gi}\right)+∋\left(X_{gi},t\right)+q_{i}\left(t\right)\phi \left(x_{gi,1},x_{gi,2},t\right)\right)\\ \; & -\sum_{j=1}^{n}a_{ij}\left(\mathrm{sat}\left(u_{gj}\right)+∋\left(X_{gj},t\right)+q_{j}\left(t\right)\phi \left(x_{gj,1},x_{gj,2},t\right)\right)\\ \; & +b_{i}\left(\mathrm{sat}\left(u_{\mathrm{gi}}\right)+∋\left(X_{gi},t\right)+q_{i}\left(t\right)\phi \left(x_{\mathrm{gi},1},\;x_{\mathrm{gi},2},t\right)\right)-b_{i}u_{0} \end{array}$$

Then, the derivative of $s_{gi}$ can be expressed as

(44) $${\dot{s}}_{gi}=K_{1}\cosh^{-2}\left(e_{gi,2}\right){\dot{e}}_{gi,2}+g_{i}\nu_{i}e_{gi,2}+g_{2}\nu_{2}e_{gi,2}+{\dot{g}}_{i}sig^{\mu_{1}}\left(e_{gi,1}\right)$$

where $\nu_{1}=\mathrm{diag}\left\{\mu_{1}{\left|e_{xgi,1}\right|}^{\mu_{1}-1},\mu_{1}{\left|e_{ygi,1}\right|}^{\mu_{1}-1}\right\}.$

(45) $$\nu_{2}=\left\{\begin{array}{c} \begin{array}{cc} \mu_{2}{\left|e_{gi,1}\right|}^{\mu_{2}-1}, & \mathrm{if}\;|e_{gi,1}|>\delta \\ a+br_{0}{\left|e_{gi,1}\right|}^{r_{0}-1}, & \mathrm{if}\;|e_{gi,1}|\leq \delta \end{array} \end{array}\right.$$

Substituting Equation (43) into Equation (44) yields

(46) $$\begin{array}{cc} {\dot{s}}_{gi} & =K_{1}{\mathrm{Cosh}}^{-2}\left(e_{gi,2}\right)\left(\left(d_{i}+b_{i}\right)\left(sat\left(u_{gi}\right)\right)-\sum_{j=1}^{n}a_{ij}\left(sat\left(u_{gj}\right)\right)\right)+K_{1}{\mathrm{Cosh}}^{-2}\left(e_{gi,2}\right)\\ & (\left(d_{i}+b_{i}\right)\left(∋\left(X_{gi},t\right)q_{i}\left(t\right)\phi \left(x_{gi,1},x_{gi,2},t\right)\right)-\sum_{j=1}^{n}a_{ij}\left(∋\left(X_{gi},t\right)+q_{j}\left(t\right)\phi \left(x_{gj,1},x_{gj,2},t\right)\right)\\ & -b_{i}u_{0})+g_{i}\nu_{1}e_{gi,2}+g_{2}\nu_{2}e_{gi,2}+{\dot{g}}_{i}sig^{\mu_{1}}\left(e_{gi,1}\right) \end{array}$$

Because $sat\left(u_{gi}\right)=u_{gi}-\Delta_{u,gi}$, $\Delta_{u,gi}$ represents the difference between the desired control input $u_{gi}$ and the actual control input $sat(u_{gi})$, where $\Delta_{u,gi}={\left[\Delta_{u,xgi},\Delta_{u,ygi}\right]}^{T}$.

So, ${\dot{s}}_{gi}$ can be organized as

(47) $$\begin{array}{cc} {\dot{s}}_{gi} & =K_{1}{\mathrm{Cosh}}^{-2}\left(e_{gi,2}\right)\left(\left(d_{i}+b_{i}\right)\left(u_{gi}\right)-\sum_{j=1}^{n}a_{ij}\left(u_{gj}\right)\right)+g_{i}\nu_{1}e_{gi,2}+g_{2}\nu_{2}e_{gi,2}\\ & +{\dot{g}}_{i}sig^{\mu_{1}}\left(e_{gi,1}\right)+{∋}_{i}^{\prime } \end{array}$$

where

$$\begin{array}{c} \begin{array}{cc} {∋}_{i}^{\prime } & =K_{1}{\mathrm{Cosh}}^{-2}\left(e_{gi,2}\right)(\left(d_{i}+b_{i}\right)\left(∋\left(X_{gi},t\right)-\Delta_{u,gi}+q_{i}\left(t\right)\phi \left(x_{gi,1},x_{gi,2},t\right)\right)\\ & -\sum_{j=1}^{n}a_{ij}\left(∋\left(X_{gj},t\right)-\Delta_{u,gj}+q_{j}\left(t\right)\phi \left(x_{gj,1},x_{gj,2},t\right)\right)-b_{i}u_{0}) \end{array} \end{array}$$

Because cosh(.)≥1, it can then be concluded that there exists an unknown normal number such that

(48) $$\begin{array}{cc} ∥{∋}^{\prime }∥ & \leq K_{1}\left\|{\mathrm{Cosh}}^{-2}\left(e_{gi,2}\right)\right\|(\left(d_{i}+b_{i}\right)\left|\right|{∋}_{i}-\Delta_{u,gi}+q_{i}\left(t\right)\phi \left(x_{gi,1},x_{gi,2},t\right)∥\\ & +\sum_{j=1}^{n}a_{ij}\|{∋}_{j}-\Delta_{u,gj}+q_{j}\left(t\right)\phi \left(x_{gj,1},x_{gj,2},t\right)\|+b_{i}\|u_{0}\left\|\right)\\ & \leq \bar{∋} \end{array}$$

The distributed adaptive sliding mode controllers are designed as follows:

(49) $$\begin{array}{cc} u_{\mathrm{gi}} & =\frac{1}{d_{i}+b_{i}}\sum_{i=1}^{n}a_{ij}\left(u_{gj}\right)-\frac{1}{K_{1}}\frac{1}{d_{i}+b_{i}}{\mathrm{Cosh}}^{2}\left(e_{gi,2}\right)\left(\alpha_{2}sig^{p_{2}}\left(s_{gi}\right)+\beta_{2}sig^{q_{2}}\left(s_{gi}\right)\right)\\ & -\frac{1}{K_{1}}\frac{1}{d_{i}+b_{i}}{\mathrm{Cosh}}^{2}\left(e_{gi,2}\right)\left({\dot{g}}_{i}sig^{\mu_{1}}\left(e_{gi,1}\right)+g_{i}\nu_{i}e_{gi,2}+g_{2}\nu_{2}e_{gi,2}\right)\\ & -\frac{1}{K_{1}}\frac{1}{d_{i}+b_{i}}{\mathrm{Cosh}}^{2}\left(e_{gi,2}\right){\hat{∋}}_{i}\frac{s_{gi}}{\left|\right|s_{gi}\left|\right|}-\frac{1}{d_{i}+b_{i}}{\hat{\phi }}_{i}^{T} \end{array}$$

The associated adaptive control law is formulated as follows:

(50) $${\dot{\hat{\bar{∋}}}}_{i}=k_{1}∥s_{gi}∥-2k_{1}k_{2}{\hat{\bar{∋}}}_{i}$$

(51) $${\dot{\hat{\phi }}}_{i}=\tau_{i}{\left(s_{gi}C\cosh^{-2}\left(e_{gi,2}\right)\right)}^{T}-\mu_{gi}{\hat{\phi }}_{i}$$

where $\alpha_{2}>0,\beta_{2}>0,p_{2}>1,0<q_{2}<1,k_{1}>0,k_{2}>0,\tau_{i}>0$, and $\mu_{\mathrm{gi}}>0$.

### 4.3. Control System

**Figure 2:**
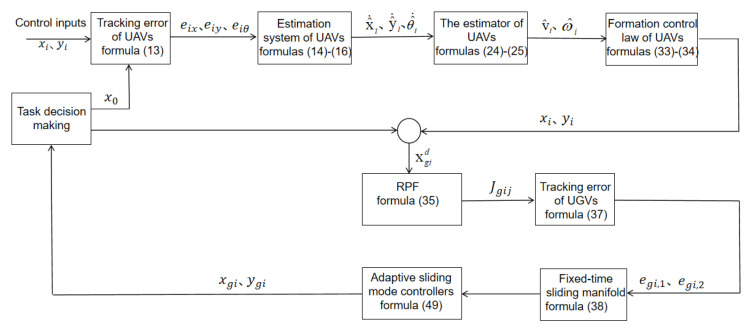
System block diagram.

The control architecture of the heterogeneous system is depicted in Figure 2. The upper section represents the UAV system, while the lower section corresponds to the UGV system.

### 4.4. Stability Analysis

**Theorem 2.**  *For system (8), $\lim_{t\to \infty }x_{ie}=0$, $\lim_{t\to \infty }y_{ie}=0$, and $\lim_{t\to \infty }\theta_{ie}=0$ in fixed time means the achievement of the control objectives of the UAVs.*

**Proof of Theorem 2.**  The Lyapunov function is introduced as

(52) $$V_{2}=\frac{\mu_{i2}}{2}\log\left(1+x_{ie}^{2}+y_{ie}^{2}\right)+\frac{\theta_{ie}^{2}}{2}$$

Taking the derivative of $V_{2}$ yields

(53) $${\dot{V}}_{2}=-\frac{\mu_{i1}\mu_{i2}x_{ie}\tanh x_{ie}}{1+x_{ie}^{2}+y_{ie}^{2}}-\mu_{i3}|\theta_{ie}{|}^{\mu_{i4}+1}$$

where $x_{ie}\tanh x_{ie}\geq 0.$

Therefore, if and only if $x_{ie}=0,y_{ie}=0$, and $\theta_{ie}=0$, this equation holds. According to Lassalle’s theorem, $\lim_{t\to \infty }\left(|x_{ie}|+|y_{ie}|+|\theta_{ie}|\right)=0$ can be obtained. Subsequently, combining Equations (26)–(28) yields $\lim_{t\to \infty }\left(|z_{ix}|+|z_{iy}|+|z_{i\theta }|\right)=0$. Therefore, the UAV system is capable of achieving the desired formation. □

In addition, because $|\tanh\left(.\right)|\leq 1,|\frac{x_{ie}^{2}}{1+x_{ie}^{2}+y_{ie}^{2}}|\leq 1$, and $|\frac{\sin\theta_{ie}}{\theta_{ie}}|\leq 1$, then there are

(54) $$|\nu_{i}|\leq |{\hat{\nu }}_{i}|+\mu_{i1}$$

(55) $$|w_{i}|\leq |{\hat{w}}_{i}|+\mu_{i2}|{\hat{\nu }}_{i}|+\mu_{i3}$$

From the above equations, it can be observed that by appropriately selecting the controller parameters $\mu_{i1}$, $\mu_{i2}$, and $\mu_{i3}$, $\nu_{i}$ and $W_{i}$ can be confined within the desired bounds.

**Theorem 3.**  *Under Assumptions 1, 2, and 4, consider the second-order MAS (11). The adaptive fixed-time controller, defined by (49) and governed by the adaptive laws (50) and (51), ensures the attainment of the control objectives for the UGV system.*

**Proof of Theorem 3.**  The Lyapunov function is introduced as

(56) $$V_{3}=\frac{1}{2}\left(\sum_{i=1}^{n}s_{gi}^{T}s_{gi}+\frac{K_{1}}{\tau_{i}}{\hat{\phi }}_{i}^{T}{\hat{\phi }}_{i}\right)+\sum_{i=1}^{n}\frac{1}{2}\frac{{\tilde{\bar{∋}}}_{i}^{2}}{k_{1}}$$

where ${\tilde{\bar{∋}}}_{i}={\bar{∋}}_{i}-{\hat{\bar{∋}}}_{i}.$

Taking the derivative of $V_{3}$ yields

(57) $$\begin{array}{cc} {\dot{V}}_{3} & =\sum_{i=1}^{n}\left(s_{gi}^{T}{\dot{s}}_{gi}+\frac{K_{1}}{\tau_{i}}{\hat{\phi }}_{i}^{T}{\dot{\hat{\phi }}}_{i}\right)-\sum_{i=1}^{n}\frac{{\tilde{\bar{∋}}}_{i}{\dot{\hat{\bar{∋}}}}_{i}}{k_{1}}\\ \; & =\sum_{i=1}^{n}s_{gi}^{T}\left({∋}_{i}^{\prime }-\left(\alpha_{2}{\mathrm{sig}}^{p_{2}}\left(s_{gi}\right)+\beta_{2}{\mathrm{sig}}^{q_{2}}\left(s_{gi}\right)\right)-{\hat{\bar{∋}}}_{i}\frac{s_{gi}}{\|s_{gi}\|}-K_{1}{\mathrm{Cosh}}^{-2}\left(e_{gi,2}\right){\hat{\phi }}_{i}^{T}\right)\\ \; & -\sum_{i=1}^{n}\frac{{\tilde{\bar{∋}}}_{i}{\dot{\hat{\bar{∋}}}}_{i}}{k_{1}}+\sum_{i=1}^{n}(K_{1}{\hat{\phi }}_{i}^{T}s_{gi}^{T}{\mathrm{Cosh}}^{-2}\left(e_{gi,2}\right)-K_{1}\frac{\mu_{gi}}{\tau_{i}}{\hat{\phi }}_{i}^{T}{\hat{\phi }}_{i}\\ \; & \leq s_{gi}^{T}\left(-\alpha_{2}\sum_{i=1}^{n}\sum_{j=1}^{m}|s_{gij}{|}^{p_{2}+1}-\beta_{2}\sum_{i=1}^{n}\sum_{j=1}^{m}|s_{gij}{|}^{q_{2}+1}\right)+\sum_{i=1}^{n}2k_{2}{\tilde{\bar{∋}}}_{i}{\hat{\bar{∋}}}_{i}-K_{1}\frac{\mu_{gi}}{\tau_{i}}\phi_{i}^{2} \end{array}$$

According to Lemma 4, the above equation can be organized as

(58) $${\dot{V}}_{3}\leq -\alpha_{2}m^{\frac{1-p_{2}}{2}}\sum_{i=1}^{n}{\left(s_{gi}^{T}s_{gi}\right)}^{\frac{P_{2}+1}{2}}-\beta_{2}\sum_{i=1}^{n}{\left(s_{gi}^{T}s_{gi}\right)}^{\frac{q_{2}+1}{2}}+\sum_{i=1}^{n}2k_{2}{\tilde{\bar{∋}}}_{i}{\hat{\bar{∋}}}_{i}-K_{1}\frac{\mu_{gi}}{\tau_{i}}{\hat{\phi }}_{i}^{2}$$

According to Yang’s inequality, it can be concluded that

(59) $$k_{2}{\tilde{\bar{∋}}}_{i}{\hat{\bar{∋}}}_{i}\leq -\frac{k_{2}\left(2o_{i}-1\right)}{2o_{i}}{\tilde{\bar{∋}}}_{i}^{2}+\frac{k_{2}o_{i}}{2}{\bar{∋}}_{i}^{2}$$

where $o_{i}>\frac{1}{2}.$

Equation (58) can be rearranged as

(60) $$\begin{array}{cc} {\dot{V}}_{3} & \leq -\alpha_{2}m^{\frac{1-p_{2}}{2}}\sum_{i=1}^{n}{\left(s_{gi}^{T}s_{gi}\right)}^{\frac{p_{2}+1}{2}}-\beta_{2}\sum_{i=1}^{n}{\left(s_{gi}^{T}s_{gi}\right)}^{\frac{q_{2}+1}{2}}-\sum_{i=1}^{n}{\left(\frac{k_{2}\left(2o_{i}-1\right)}{2o_{i}}{\tilde{\bar{∋}}}_{i}^{2}\right)}^{\frac{p_{2}+1}{2}}\\ \; & -{\left(\frac{k_{2}\left(2o_{i}-1\right)}{2o_{i}}{\tilde{\bar{∋}}}_{i}^{2}\right)}^{\frac{q_{2}+1}{2}}+\sum_{i=1}^{n}{\left(\frac{k_{2}\left(2o_{i}-1\right)}{2o_{i}}{\tilde{\bar{∋}}}_{i}^{2}\right)}^{\frac{p_{2}+1}{2}}+\sum_{i=1}^{n}{\left(\frac{k_{2}\left(2o_{i}-1\right)}{2o_{i}}{\tilde{\bar{∋}}}_{i}^{2}\right)}^{\frac{q_{2}+1}{2}}\\ \; & -\sum_{i=1}^{n}\frac{k_{2}\left(2o_{i}-1\right)}{o_{i}}{\tilde{\bar{∋}}}_{i}^{2}+\sum_{i=1}^{n}k_{2}o_{i}{\bar{∋}}_{i}^{2}-K_{1}\frac{\mu_{gi}}{\tau_{i}}{\hat{\phi }}_{i}^{2} \end{array}$$

Because ${\left(\frac{k_{2}\left(2o_{i}-1\right)}{2o_{i}}{\tilde{\bar{∋}}}_{i}^{2}\right)}^{\frac{q_{2}+1}{2}}-\frac{k_{2}\left(2o_{i}-1\right)}{2o_{i}}{\tilde{\bar{∋}}}_{i}^{2}\leq 1$, Equation (60) can be summarized as

(61) $$\begin{array}{cc} {\dot{V}}_{3} & \leq -\alpha_{2}m^{\frac{1-p_{2}}{2}}{\left(s_{gi}^{T}s_{gi}\right)}^{\frac{P_{2}+1}{2}}-\beta_{2}{\left(s_{gi}^{T}s_{gi}\right)}^{\frac{q_{2}+1}{2}}-{\left(\frac{k_{2}\left(2o_{i}-1\right)}{2o_{i}}{\tilde{\bar{∋}}}_{i}^{2}\right)}^{\frac{p_{2}+1}{2}}-{\left(\frac{k_{2}\left(2o_{i}-1\right)}{2o_{i}}{\tilde{\bar{∋}}}_{i}^{2}\right)}^{\frac{q_{2}+1}{2}}\\ \; & +{\left(\frac{k_{2}\left(2o_{i}-1\right)}{2o_{i}}{\tilde{\bar{∋}}}_{i}^{2}\right)}^{\frac{p_{2}+1}{2}}-\frac{k_{2}\left(2o_{i}-1\right)}{2o_{i}}{\tilde{\bar{∋}}}_{i}^{2}+k_{2}o_{i}{\bar{∋}}_{i}^{2}+1-K_{1}\frac{\mu_{gi}}{\tau_{i}}{\hat{\phi }}_{i}^{2} \end{array}$$

Assume η>0 and $|{\tilde{\bar{∋}}}_{i}|\leq \eta_{i}$.

If $\eta_{i}<\sqrt{\frac{2o_{i}}{k_{2}\left(2o_{i}-1\right)}}$, then $\sum_{i=1}^{n}{\left(\frac{k_{2}\left(2o_{i}-1\right)}{2o_{i}}{\tilde{\bar{∋}}}_{i}^{2}\right)}^{\frac{p_{2}+1}{2}}<\sum_{i=1}^{n}\frac{k_{2}\left(2o_{i}-1\right)}{2o_{i}}{\tilde{\bar{∋}}}_{i}^{2}$;

If $\eta_{i}\geq \sqrt{\frac{2o_{i}}{k_{2}\left(2o_{i}-1\right)}}$, then $\sum_{i=1}^{n}{\left(\frac{k_{2}\left(2o_{i}-1\right)}{2o_{i}}{\tilde{\bar{∋}}}_{i}^{2}\right)}^{\frac{p_{2}+1}{2}}-\sum_{i=1}^{n}\frac{k_{2}\left(2o_{i}-1\right)}{2o_{i}}{\tilde{\bar{∋}}}_{i}^{2}\leq \sum_{i=1}^{n}{\left(\frac{k_{2}\left(2o_{i}-1\right)}{2o_{i}}\eta_{i}^{2}\right)}^{\frac{p_{2}+1}{2}}$ $-\sum_{i=1}^{n}\frac{k_{2}\left(2o_{i}-1\right)}{2o_{i}}\eta_{i}^{2}$.

Equation (61) can be rearranged as

(62) $$\begin{array}{cc} {\dot{V}}_{3} & \leq -\alpha_{2}m^{\frac{1-p_{2}}{2}}{\left(s_{gi}^{T}s_{gi}\right)}^{\frac{P_{2}+1}{2}}-\beta_{2}{\left(s_{gi}^{T}s_{gi}\right)}^{\frac{q_{2}+1}{2}}-{\left(\frac{k_{2}\left(2o_{i}-1\right)}{2o_{i}}{\tilde{\bar{∋}}}_{i}^{2}\right)}^{\frac{p_{2}+1}{2}}-{\left(\frac{k_{2}\left(2o_{i}-1\right)}{2o_{i}}{\tilde{\bar{∋}}}_{i}^{2}\right)}^{\frac{q_{2}+1}{2}}\\ \; & +{\left(\frac{k_{2}\left(2o_{i}-1\right)}{2o_{i}}\eta_{i}^{2}\right)}^{\frac{p_{2}+1}{2}}-\frac{k_{2}\left(2o_{i}-1\right)}{2o_{i}}\eta_{i}^{2}+k_{2}o_{i}{\bar{∋}}_{i}^{2}+1-K_{1}\frac{\mu_{gi}}{\tau_{i}}{\hat{\phi }}_{i}^{2} \end{array}$$

Therefore, from Lemma 3, it can be concluded that there exist two positive constants α and β such that

(63) $${\dot{V}}_{3}\leq -\alpha V_{2}^{\frac{p_{2}-1}{2}}-\beta V_{2}^{\frac{q_{2}-1}{2}}+\zeta$$

where $\zeta ={\left(\frac{k_{2}\left(2o_{i}-1\right)}{2o_{i}}\eta_{i}^{2}\right)}^{\frac{p_{2}+1}{2}}-\frac{k_{2}\left(2o_{i}-1\right)}{2o_{i}}\eta_{i}^{2}+k_{2}o_{i}{\bar{∋}}_{i}^{2}+1-K_{1}\frac{\mu_{gi}}{\tau_{i}}{\hat{\phi }}_{i}^{2}$.

From Equation (63), the bounded convergence region of $s_{gi}$ is guaranteed, which is defined as

(64) $$\Omega_{s_{gi}}=\left\{s_{gi}|V_{2}\left(x\right)\leq \min\left\{{\left(\frac{\zeta }{(1-\varpi )\alpha }\right)}^{\frac{1}{P_{2}}},{\left(\frac{\zeta }{(1-\varpi )\beta }\right)}^{\frac{1}{q_{2}}}\right\}\right\}$$

System (11) is proven to stabilize at the origin under a fixed convergence time constraint, where the fixed time is given by

(65) $$T_{3}\leq \frac{1}{\alpha \varpi (p_{2}-1)}+\frac{1}{\beta \varpi (1-q_{2})}$$

□

Based on its fixed-time stability expression, the system can achieve stabilization within an adjustable time frame, enabling followers to track the leader UAV and complete specific formation configurations.

## 5. Simulation

### 5.1. Heterogeneous Simulation

To verify the effectiveness of Equations (33), (34) and (49), simulation experiments were performed on the UAV-UGV system. The heterogeneous MAS consisted of six UAVs and three UGVs. The agents’ positions and parameters of the agents are detailed in Table 1, Table 2 and Table 3.

**Figure 3:**
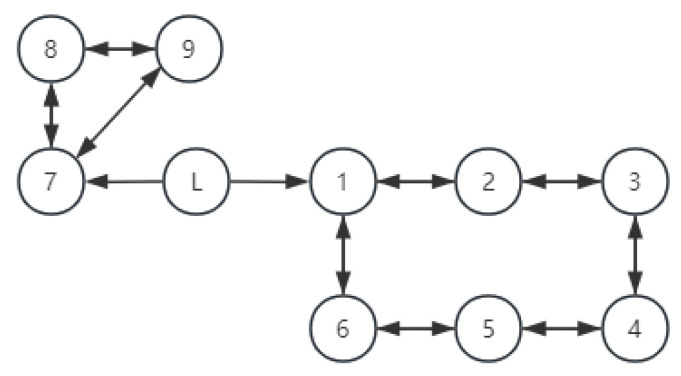
Communication topology.

Figure 3 illustrates the communication topology, agent 2–agent 7 were UAVs, and agent 8–agent 10 were UGVs. Under the proposed protocol, the system was required to achieve formation within a fixed settling time.

We assumed the desired formation for multiple agents was a triangle, with the desired positional deviations defined as $\Delta_{1}={\left[\begin{array}{c} 4,4 \end{array}\right]}^{T}$, $\Delta_{2}={\left[\begin{array}{c} -4,4 \end{array}\right]}^{T}$, and $\Delta_{3}={\left[\begin{array}{c} 0,-4 \end{array}\right]}^{T}$. ${∋}_{i},i=1,2,3$ were defined as ${∋}_{1}=0.02{\left[\sin(0.4t),\cos(0.4t)\right]}^{T}$, ${∋}_{2}=0.01{\left[\sin(0.8t),\cos(0.4t)\right]}^{T}$, and ${∋}_{3}=0.006{\left[\sin(0.2t),\cos(0.3t)\right]}^{T}$. The actuator attacks were modeled as $\phi (x_{gi,1},x_{gi,2},t)$ $=0.5\sin(x_{gi,1}\left(t\right)+x_{gi,2}\left(t\right))$. The external disturbances that acted on UAV2 were modeled as $d_{2}=0.1\cos\left(0.1t\right)$. The external disturbances that acted on UAV4 were modeled as $d_{4}=0.5\sin\left(0.1t\right)$.

**Figure 4:**
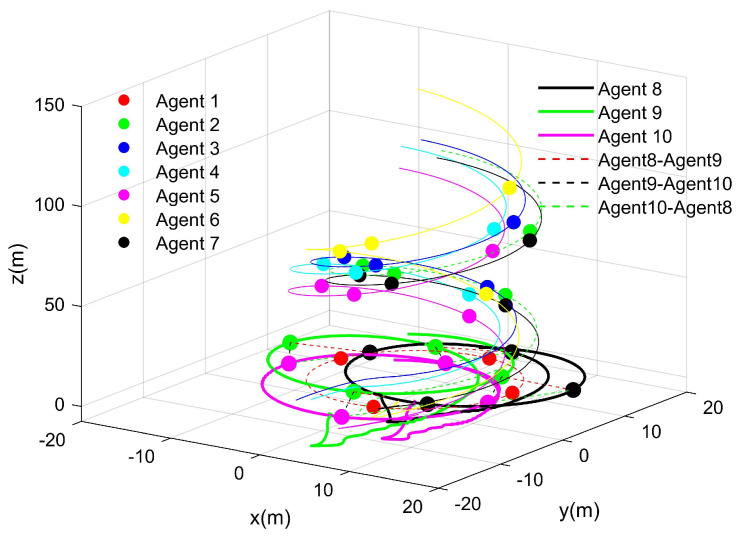
Agent trajectories.

The simulation result is shown in Figure 4.

According to Figure 4, which shows the simulation results, under external disturbances, the proposed fixed-time distributed control protocol ensured tracking error convergence for the UAVs and UGVs, which enabled them to achieve the desired formation within the predefined time frame. This demonstrates the protocol’s robustness and consensus-based coordination in complex environments with disturbances.

**Figure 5:**
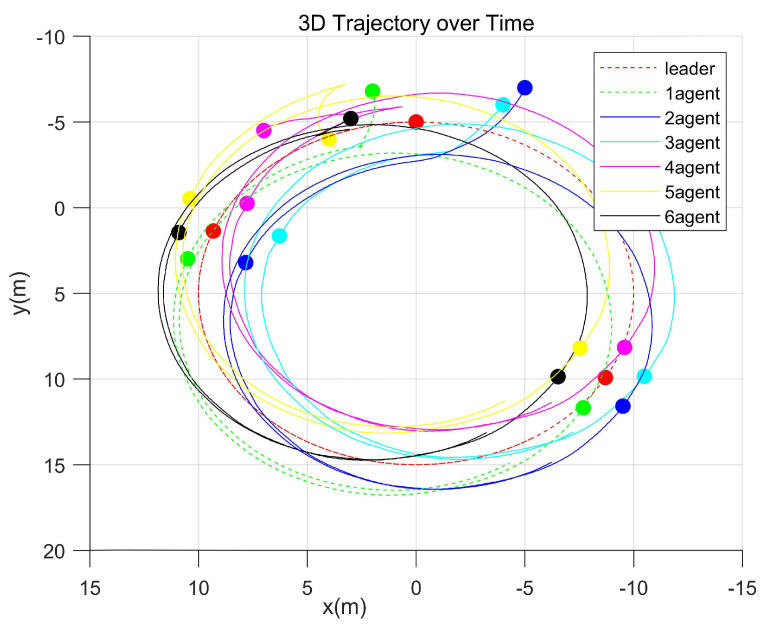
UAV trajectories.

By breaking down Figure 4, the trajectory path corresponding to each UAV is clearly depicted, as demonstrated in Figure 5.

Figure 5 illustrates the tracking performance of the follower UAVs, where the red dashed curve represents the desired trajectory of the virtual leader, and the six other colored curves correspond to the actual trajectories of the follower UAVs. Additionally, the configurations at three distinct times—initial state, pre-formation, and post-formation—are plotted. From this simulation, it is evident that under the proposed control protocol, the formation system rapidly overcame the initial errors, achieved the desired formation, and tracked the reference trajectory with a high precision.

**Figure 6:**
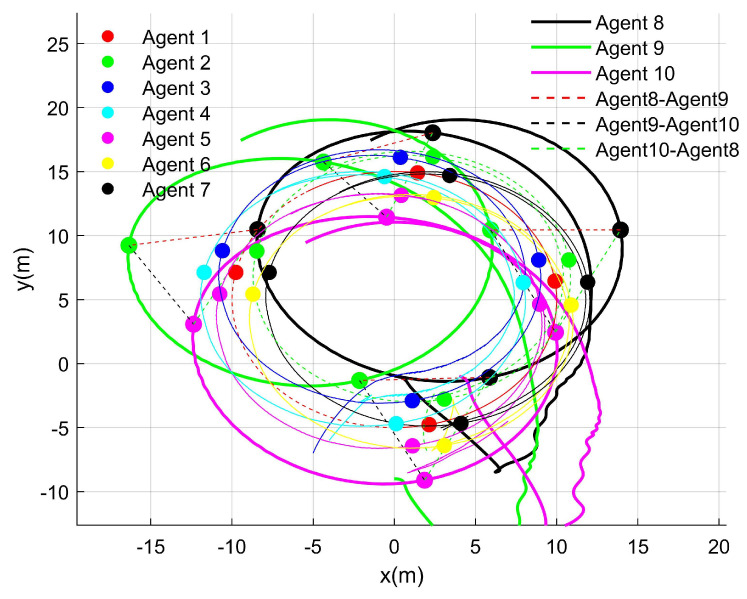
Top-down view of the heterogeneous multi-agent system.

Figure 6 presents a top-down view of the heterogeneous system, where hollow circles represent the positions of the UGVs. The dashed lines connecting these hollow circles clearly illustrate the triangular formation of the system, highlighting the geometric configuration achieved through the proposed control strategy.

Figure 6 illustrates four distinct states of the UGVs and UAVs during both the initialization and operation. It clearly demonstrates that the UGVs and UAVs maintained their respective formations while following the virtual leader in a coordinated multi-agent framework.

**Figure 7:**
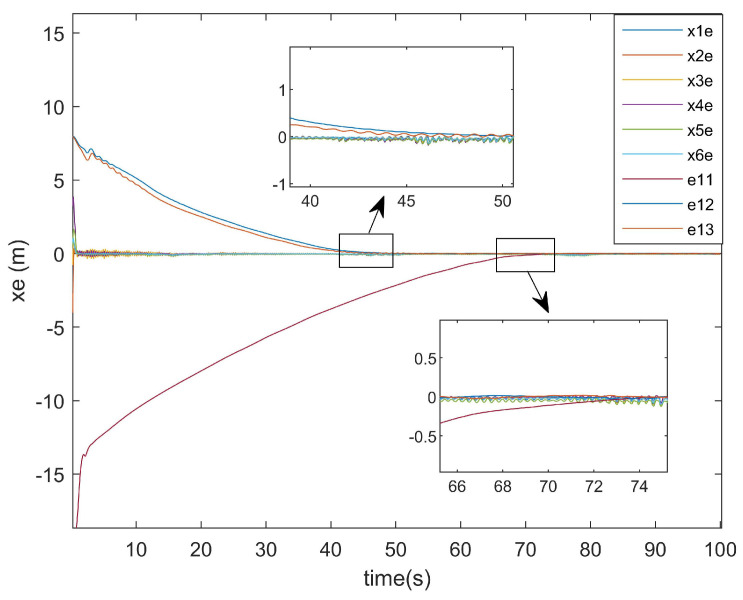
*x*-axis error.

**Figure 8:**
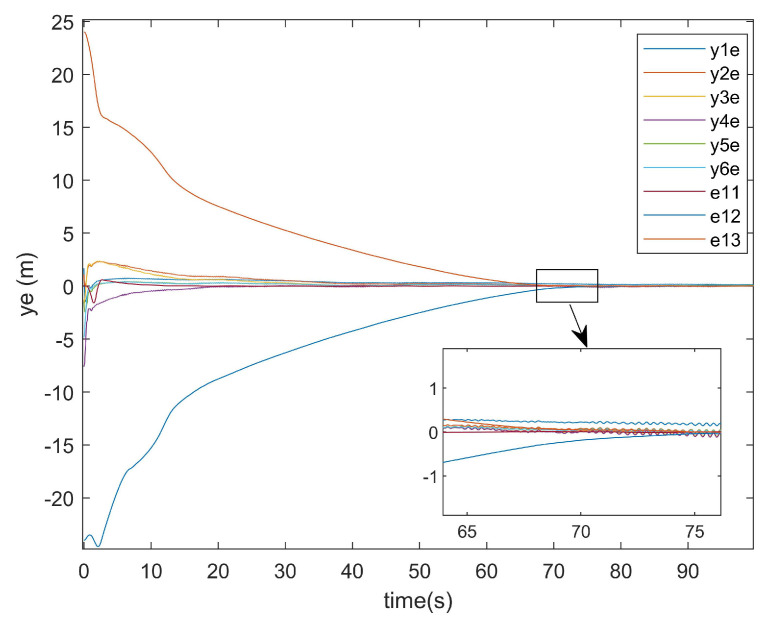
y-axis error.

The position error diagrams are shown in Figure 7 and Figure 8, demonstrating the trajectory tracking performance in multi-agent formation control systems.

As shown in Figure 7 and Figure 8, the convergence time of the UGVs system was approximately 72 s. This delay arose from the formation tracking control requirements under multiple constraints, including input saturation, external disturbances, and actuator attacks, which collectively impeded the convergence rate. Despite the presence of initial tracking errors, the system achieved asymptotic stability by maintaining the tracking errors within a bounded and stable range, demonstrating robustness against the aforementioned constraints.

### 5.2. Simulation Comparison

To demonstrate the superiority of the proposed protocol, this section describes comparative simulation experiments with reference [11] (the study that only considered scenarios with external conditions/external interference).

**Figure 9:**
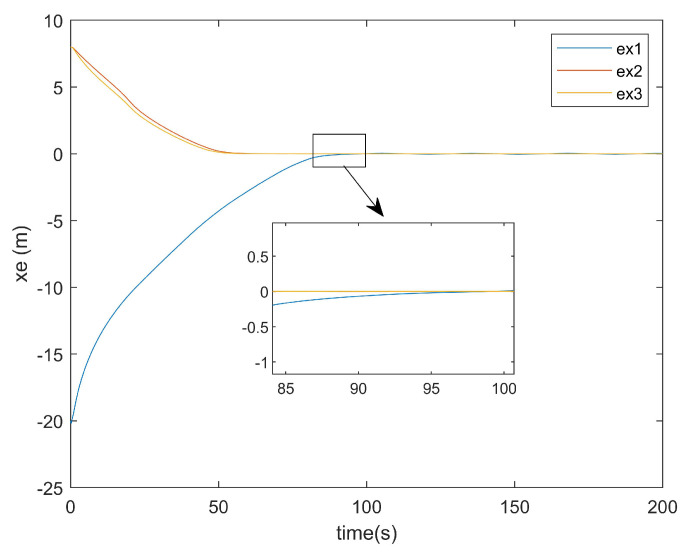
*x*-axis error plot of existing method.

**Figure 10:**
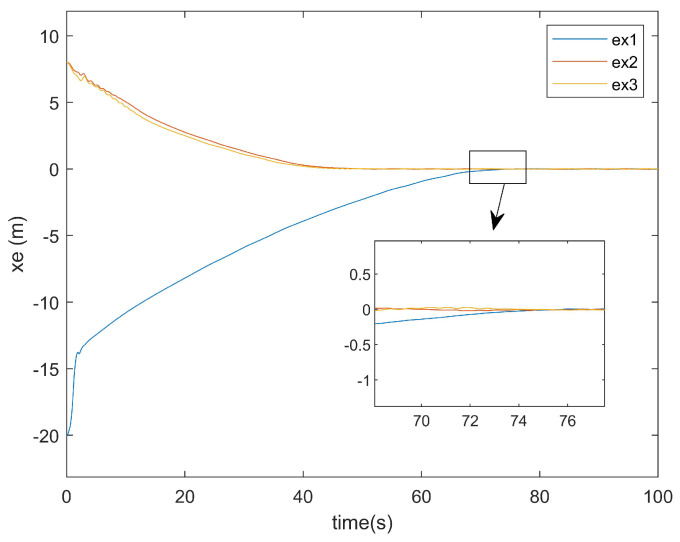
*x*-axis error plot of this article.

Regarding the position error of the *x*-axis, Figure 9 illustrates the *x*-axis error plot of the existing method, while Figure 10 presents the *x*-axis error plot of the approach proposed in this paper.

As shown in Figure 9, the existing method required approximately 95 s to achieve *x*-axis tracking. In contrast, Figure 10 reveals that the method proposed in this paper accomplished the *x*-axis tracking in only around 75 s. Notably, despite incorporating two additional factors—input saturation and actuator faults—the convergence time was reduced by 20 s. This demonstrates the superior convergence effectiveness of the proposed controller at addressing *x*-axis positioning errors.

**Figure 11:**
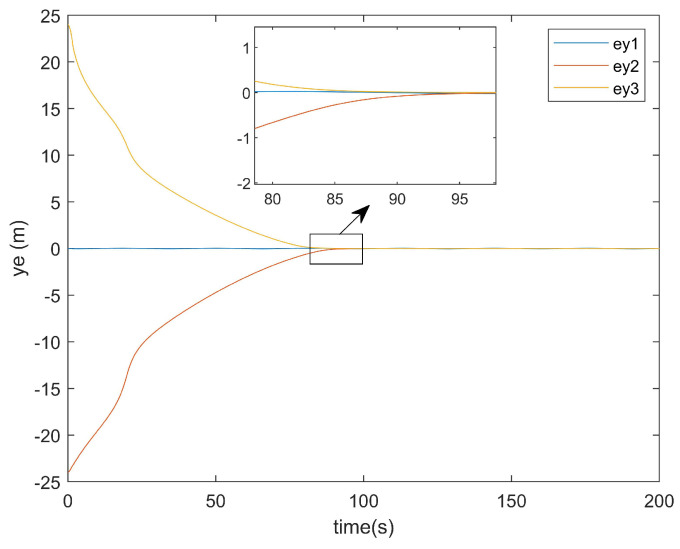
*y*-axis error plot of existing method.

**Figure 12:**
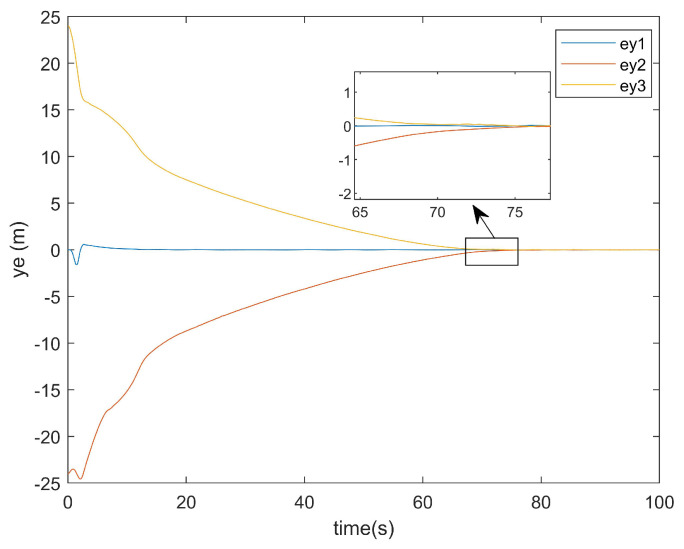
The *y*-axis error plot of this article.

For the positional error along the *y*-axis, Figure 11 shows the *y*-axis error graph of the existing method, while Figure 12 displays the *y*-axis error graph presented in this paper.

As can be seen from Figure 11, the existing method exhibited a tracking time along the *y*-axis that was very close to that along the *x*-axis. Similarly, Figure 12 demonstrates that the method presented in this paper also achieved a *y*-axis tracking time comparable to its *x*-axis counterpart.

Overall, compared with the existing method, the controller proposed in this paper additionally considers both input saturation and actuator failures while achieving a 20-s faster error convergence time. This demonstrates that the controller developed in this study achieved superior convergence performance.

**Figure 13:**
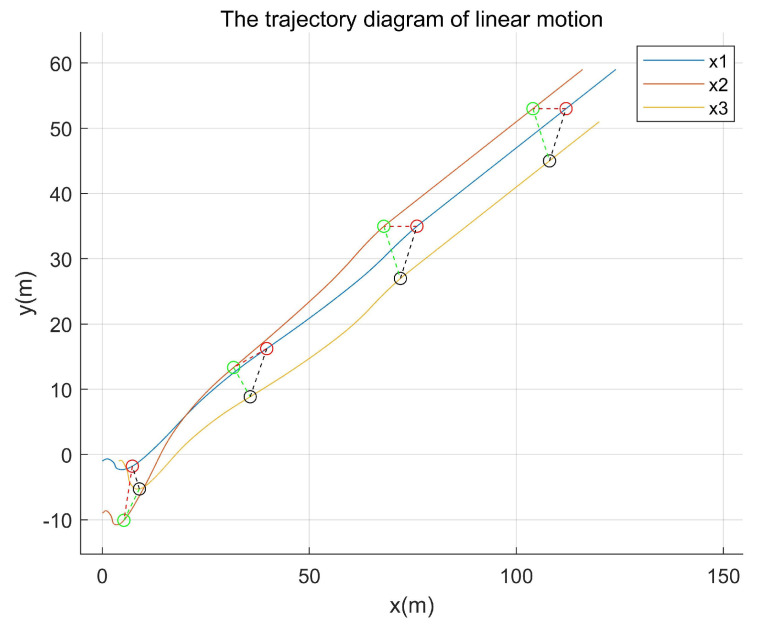
The trajectory path diagram of the existing method.

**Figure 14:**
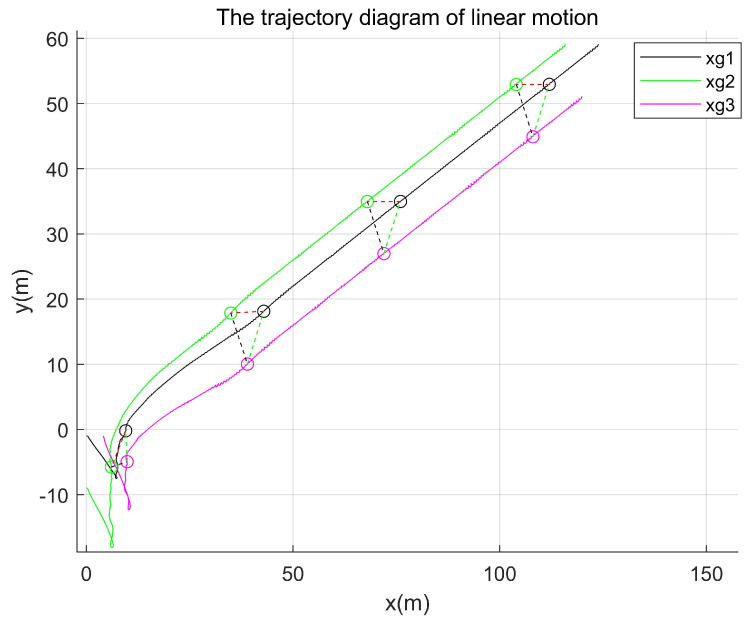
Trajectory path diagram of the proposed method.

Comparative experiment 2: By changing the leader’s trajectory to straight-line motion, the trajectory path diagram of the existing method is shown in Figure 13, while that of the proposed method in this paper is presented in Figure 14.

As shown in both Figure 13 and Figure 14, the initial velocity and starting positions of the agents, as well as the time points corresponding to their positions depicted in the simulation, were maintained. As demonstrated in Figure 13 and Figure 14, the method proposed in this paper required a shorter formation time and achieved the target formation pattern more rapidly.

**Figure 15:**
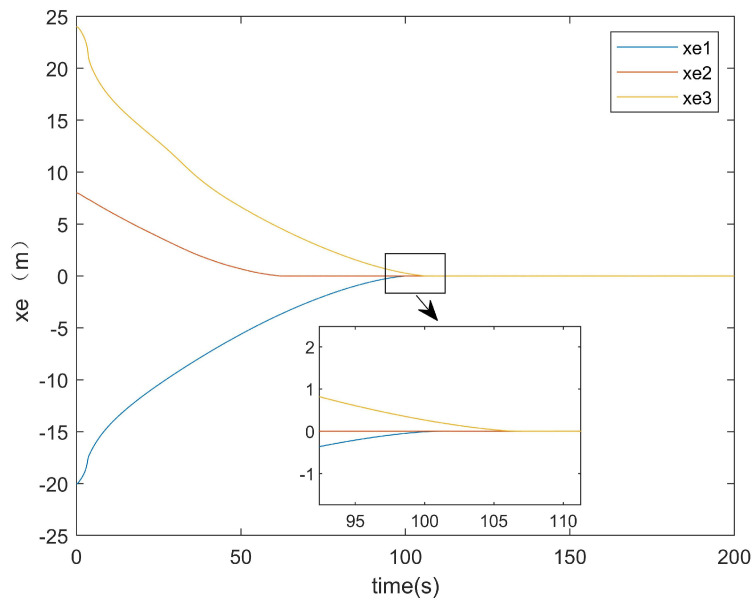
*x*-axis error of the conventional method.

**Figure 16:**
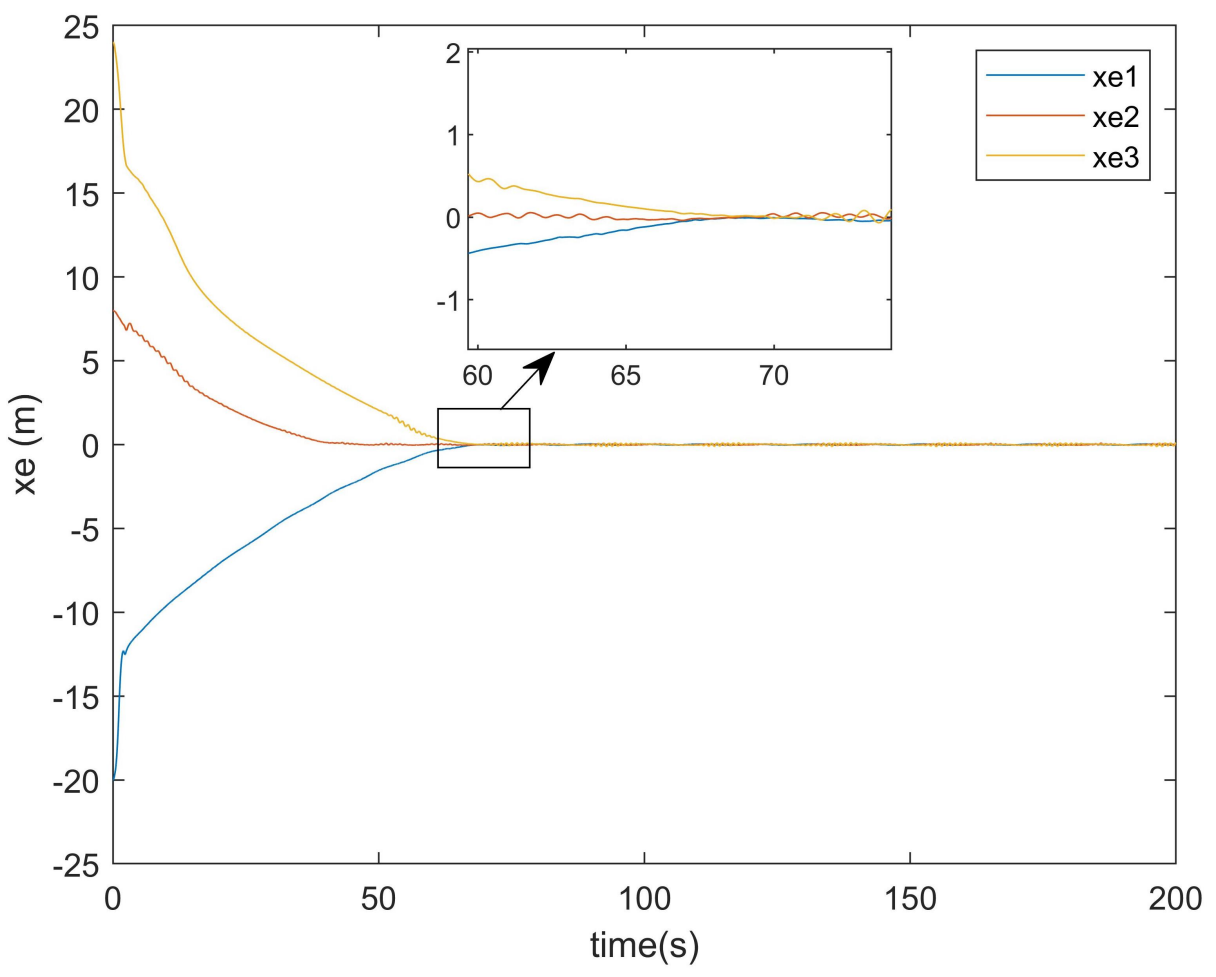
*x*-axis error of the proposed method.

Subsequently, the error convergence times of the two methods were compared. Figure 15 presents the *x*-axis error convergence diagram of the conventional method, while Figure 16 illustrates that of the proposed method.

It can be seen from Figure 15 that the convergence of the error on the *x*-axis for the existing method took about 108 s. From Figure 16, it can be observed that the convergence of the error on the *x*-axis for the method proposed in this paper only took about 70 s. From the locally enlarged view, it can be seen that there was still an error after tracking. This was because the situations of actuator attacks and input saturation were considered in this paper, and there was an error of approximately 0.1 in the error curve after convergence. It can be concluded that the convergence time of the error on the *x*-axis for the method in this paper was about 30 s faster than that of the existing method for the error on the *x*-axis.

**Figure 17:**
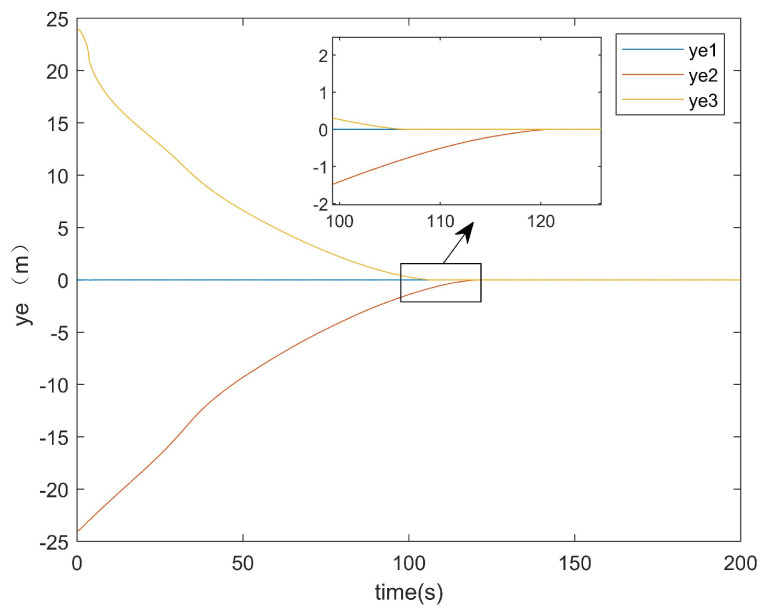
*y*-axis error of the conventional method.

**Figure 18:**
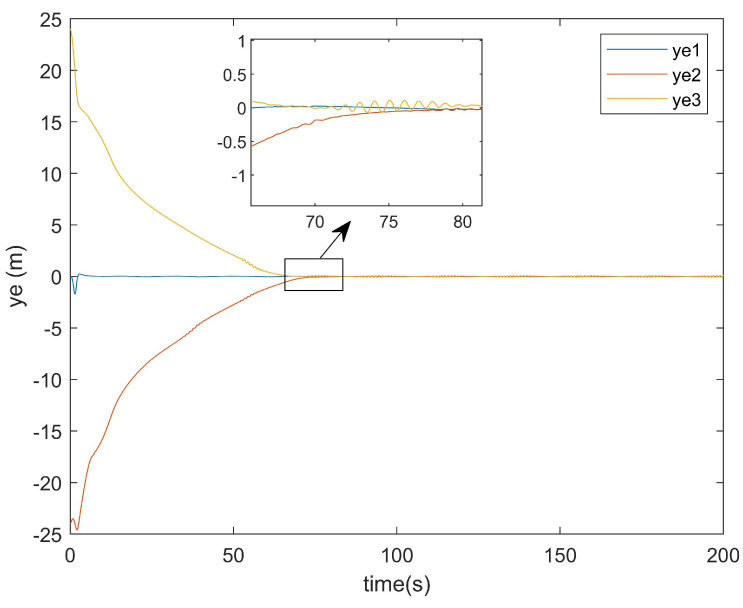
*y*-axis error of the proposed method.

Figure 17 presents the *y*-axis error convergence diagram of the conventional method, while Figure 18 illustrates that of the proposed method.

As demonstrated in Figure 17, the conventional method required approximately 120 s to achieve the *y*-axis error convergence, whereas the proposed method attained convergence within only 80 s (Figure 18). This represents a 40-s improvement in the *y*-axis error convergence time compared with the conventional method.

As evidenced by the two comparative experiments above, the proposed controller not only addressed additional constraints (e.g., actuator attacks and input saturation) compared with the original disturbance rejection controller but also achieved faster convergence rates, which resulted in significantly shorter formation times.

## 6. Conclusions

This paper addresses the formation tracking control of heterogeneous MASs under the simultaneous consideration of input saturation, actuator attacks, and external disturbances. For such systems, a distributed sliding mode formation tracking control protocol was designed by integrating a fixed-time control algorithm and an adaptive sliding mode control method. To address collision avoidance between agents, the RPF was incorporated into the sliding manifold, enabling the system to track the virtual leader under fixed and adjustable time intervals while ensuring collision-free formation under fixed interaction topologies.

Finally, numerical simulations were simulated in MATLAB2021b. The simulation outcomes confirmed that the proposed controller enabled the follower UAV-UGV to acquire the virtual leader’s state information and achieve collision-free formation tracking within fixed and adjustable time intervals, validating the efficacy of the distributed adaptive sliding mode control protocol under heterogeneous multi-agent dynamics.

In the current study, the theoretical framework and simulation verification of the control strategy were primarily emphasized, which led to limited exploration of its limitations and real-world challenges. Future investigations should prioritize practical implementation challenges, including collision avoidance for UAV swarms, obstacle negotiation for UGV formations, and heterogeneous communication latency. Systematic resolution of these technical barriers is essential to strengthen the practical applicability of the multi-agent framework in operational scenarios.

## Figures and Tables

**Table 1:** Initial positions.

Agent	Value	Agent	Value
Leader	${\left[0,-5,0\right]}^{T}$	UAV1	${\left[1,\sqrt{3},0\right]}^{T}$
UAV2	${\left[-1,\sqrt{3},0\right]}^{T}$	UAV3	${\left[-2,0,0\right]}^{T}$
UAV4	${\left[-1,-\sqrt{3},0\right]}^{T}$	UAV5	${\left[1,-\sqrt{3},0\right]}^{T}$
UAV6	${\left[2,0,0\right]}^{T}$	UAV7	${\left[0,-1,0\right]}^{T}$
UAV8	${\left[0,-9,0\right]}^{T}$	UAV9	${\left[4,-1,0\right]}^{T}$

**Table 2:** UAV parameters.

Parameter	Value	Parameter	Value
$C_{X}$	3	$C_{y}$	2
$C_{\theta }$	2.2	$D_{X}$	0.5
$D_{Y}$	0.5	$D_{\theta }$	1.2
$\mu_{i1}$	18	$\mu_{i2}$	0.2
$\mu_{i3}$	1.5		

**Table 3:** UGV parameters.

Parameter	Value	Parameter	Value
$K_{1}$	8	$R_{out}$	6
$K_{2}$	6	$R_{in}$	3
$m_{1}$	21	$\rho_{12},\rho_{13},\rho_{23}$	0.001
$n_{1}$	14	$\alpha_{2}$	2
$m_{2}$	1.5	$\beta_{2}$	1
$n_{2}$	3.8	$p_{2}$	1.1
$r_{0}$	2	$q_{2}$	1/3
$g_{0}$	0.01	$k_{1},k_{2},k_{3}$	5

## Data Availability

Data are contained within the article.
